# Curcumin Attenuates LPS-Induced Migration/EMT and LPS/ATP-Associated IL-1β Release in Androgen-Independent Prostate Cancer Cells

**DOI:** 10.3390/cimb48040413

**Published:** 2026-04-17

**Authors:** Mon-Der Cho, Shang-Yu Chou, Yu-Ming Hsu, Chi-Ying Li, Yi-Hong Tsai, Fang-Rong Chang

**Affiliations:** 1 Graduate Institute of Natural Products, Kaohsiung Medical University, Kaohsiung 807378, Taiwan; u105831003@kmu.edu.tw; 2 Department of Urology, Kaohsiung Municipal Min-Sheng Hospital, Kaohsiung 802511, Taiwan; 3 Department of Radiation Oncology, Kaohsiung Chang Gung Memorial Hospital, Kaohsiung 824005, Taiwan; a9682@adm.cgmh.org.tw; 4 Department of Pharmacy and Master Program, College of Pharmacy and Health Care, Tajen University, Pingtung County 907101, Taiwan; maiz538272@gmail.com; 5 Research Center for Precision Environmental Medicine, Kaohsiung Medical University, Kaohsiung 807378, Taiwan; 6 School of Pharmacy, Tzu Chi University, Hualien 970374, Taiwan; cyli@gms.tcu.edu.tw; 7 Department of Marine Biotechnology and Resources, National Sun Yat-sen University, Kaohsiung 804201, Taiwan; 8 Drug Development and Value Creation Research Center, Kaohsiung Medical University, Kaohsiung 807378, Taiwan; 9 Department of Medical Research, Kaohsiung Medical University Hospital, Kaohsiung Medical University, Kaohsiung 807378, Taiwan

**Keywords:** prostate cancer, curcumin, lipopolysaccharide (LPS), epithelial–mesenchymal transition (EMT), reactive oxygen species (ROS), NLRP3 inflammasome

## Abstract

Inflammation can promote aggressive phenotypes in prostate cancer, including enhanced migration/EMT-like changes and inflammasome-associated cytokine release. Here, we examined whether curcumin modulates these inflammation-driven responses in androgen-independent prostate cancer cells. PC-3 and DU145 cells were treated with curcumin (10 or 25 μM) or N-acetylcysteine (NAC; 2 mM). Sub-cytotoxic dosing was defined by CCK-8 viability assays. LPS (0.5 μg/mL) was used to induce motility-, invasion-, and EMT-associated responses, assessed by wound-healing assay, Matrigel-coated Transwell invasion assay, and RT–qPCR of *SNAI1*, *CDH1*, and *VIM*. Intracellular ROS was quantified by CM-H_2_DCFDA flow cytometry. Inflammasome-associated and EMT-related protein changes were evaluated under LPS priming (24 h) followed by ATP triggering (5 mM, 1 h), with NLRP3, cleaved caspase-1, cleaved IL-1β, vimentin, and E-cadherin assessed by immunoblotting and IL-1β secretion measured by ELISA. Curcumin at 10–25 μM did not cause overt cytotoxicity and significantly reduced LPS-induced wound closure and invasive activity in both cell lines, accompanied by attenuation of EMT-associated transcriptional changes and a decrease in ROS-positive events. Under LPS priming/ATP triggering, inflammasome-associated protein signals and IL-1β secretion were robustly induced; curcumin suppressed IL-1β release and attenuated NLRP3, cleaved caspase-1, and cleaved IL-1β signals, while reversing vimentin/E-cadherin changes. NAC produced similar inhibitory patterns, supporting a redox-linked contribution to these responses. Collectively, curcumin dampens inflammation-driven motility/invasion, EMT-associated changes, and inflammasome-associated responses in androgen-independent prostate cancer cells.

## 1. Introduction

Prostate cancer is a growing global health challenge. The Lancet Commission projects that the number of new cases will rise from 1.4 million in 2020 to 2.9 million by 2040 [1]. Although many localized tumors can be treated with curative intent, advanced disease often progresses despite androgen-deprivation therapy and ultimately becomes castration-resistant and largely incurable [2,3]. The major cause of death is metastatic spread; in a large SEER-based cohort, 77.8% of deaths among men with metastatic prostate cancer were directly attributable to the disease [4]. Despite recent therapeutic advances, drug resistance and subsequent progression remain common in advanced stages [5,6]. Together, these observations highlight an urgent need for new mechanistic targets and strategies to prevent or delay lethal progression.

Chronic inflammation is increasingly viewed as an important feature of the prostate microenvironment and a contributor to tumor-promoting conditions. Persistent inflammatory mediators and immune–epithelial interactions can reshape local tissue homeostasis, enriching the milieu with cytokines, chemokines, and growth factors that support epithelial remodeling and malignant progression [7]. Bacterial lipopolysaccharide (LPS) is a well-established experimental stimulus of TLR4 signaling. Although not a dominant physiological driver in most prostate cancer settings, it is commonly used to model inflammation-associated signaling in cancer cells. Accordingly, LPS was used here as a defined inflammatory stimulus rather than as the predominant in vivo driver of disease progression [8]. TLR4 activation engages downstream pathways, including NF-κB-linked transcriptional programs, which have been associated with pro-tumor behaviors [7]. In prostate cancer models, elevated TLR4 signaling has been reported to correlate with increased proliferation and enhanced migratory/invasive capacity. In addition, LPS-driven signaling has been implicated in EMT-related transcriptional changes in prostate epithelial/cancer contexts. Together, these findings provide a rationale to examine migration and EMT-associated gene expression as functional readouts of inflammation-driven plasticity in prostate cancer [9].

Epithelial–mesenchymal transition (EMT) is a key program that enables cancer cells to become more mobile and invasive. During EMT, cells lose epithelial features, such as cell–cell junctions, and gain the ability to migrate and invade surrounding tissues [10]. This shift is typically marked by decreased E-cadherin (CDH1) and increased vimentin (VIM) and is driven in part by EMT transcription factors such as the Snail family, which can repress E-cadherin and promote mesenchymal gene expression [11]. EMT-like changes are therefore often linked to metastatic spread, in which tumor cells acquire enhanced motility and invasiveness [12].

In prostate cancer, inflammatory cues may intersect with this EMT–migration axis. LPS-driven signaling has been reported to activate NF-κB and promote EMT-related changes in prostate epithelial/cancer models, and TLR4 (a major LPS receptor) has been associated with aggressive features; blocking TLR4 signaling can reduce prostate cancer cell proliferation and migration in vitro [13,14]. Despite these observations, it remains unclear how LPS-related inflammatory stimulation connects EMT-associated transcriptional changes (e.g., SNAI1, CDH1, and VIM) to measurable migratory behavior in androgen-independent prostate cancer cells [15]. This gap provides a clear rationale for examining migration and EMT readouts side by side in our model.

Inflammatory stimulation often leads to increased reactive oxygen species (ROS). Beyond being stress byproducts, ROS function as signaling molecules that influence pathways involved in inflammation and cancer cell behavior, including migration [16]. Oxidative stress has also been linked to EMT, a process typically marked by loss of epithelial features (e.g., reduced E-cadherin) and gain of mesenchymal traits (e.g., increased vimentin), which together support greater motility and invasiveness [17]. Importantly, LPS signaling can promote ROS generation through TLR4-dependent activation of NADPH oxidases, providing a plausible route by which inflammatory cues reshape intracellular redox status [18,19]. Based on this framework, we hypothesized that LPS-induced ROS accumulation may connect inflammatory stimulation to EMT-associated transcriptional changes and enhanced migration. We therefore included N-acetylcysteine (NAC) as a redox-modulating comparator to probe the contribution of oxidative stress in this model.

The NLRP3 inflammasome is a key innate immune platform that senses microbial or danger signals and forms a multiprotein complex that activates caspase-1, enabling maturation and release of pro-inflammatory cytokines such as IL-1β and IL-18 [20]. Given its well-characterized role as a functional inflammasome output, IL-1β release is often used as a practical readout of inflammasome-associated responses. Canonical NLRP3 activation is often described as a two-step process: priming (signal 1), commonly triggered by ligands such as LPS, which increases expression of inflammasome components and pro-IL-1β; and activation (signal 2), in which stimuli such as extracellular ATP promote inflammasome assembly and cytokine processing [21]. Beyond host defense, inflammasome-derived cytokines—especially IL-1β—have been linked to tumor-supporting inflammation in some settings, influencing cancer cells and the surrounding microenvironment [22].

Curcumin is a natural polyphenol with reported anti-inflammatory and antioxidant activities and broad effects on cellular signaling [23,24]. It has been linked to activation of the Nrf2 antioxidant program and modulation of redox- and inflammation-related pathways, including NF-κB, NOX, and MAPK, suggesting potential to limit oxidative stress and inflammatory responses [25,26]. Prior studies also indicate that curcumin can interfere with inflammasome-related pathways, including NLRP3-associated caspase-1 activation and IL-1β production in immune-cell models [27].

Despite this background, its role in inflammation-driven prostate cancer phenotypes remains incompletely defined. Inflammatory cues such as LPS can promote a more migratory, EMT-like state, yet it is unclear whether curcumin can suppress LPS-induced migration together with EMT-associated transcriptional changes in androgen-independent prostate cancer cells [28]. In addition, although LPS priming followed by ATP stimulation is a classical approach to trigger inflammasome-related outputs, whether curcumin can dampen this response in prostate cancer cells—particularly IL-1β secretion—remains unclear.

Based on these considerations, we hypothesized that curcumin attenuates inflammation-driven aggressive phenotypes in androgen-independent prostate cancer cells by suppressing migration- and EMT-associated changes and by limiting inflammasome-associated inflammatory responses. Therefore, the aim of this study was to investigate whether curcumin modulates LPS-induced changes in motility and EMT-related markers, oxidative stress, and LPS-primed/ATP-triggered inflammasome-associated responses in PC-3 and DU145 cells.

## 2. Materials and Methods

### 2.1. Materials

Cell-culture-related reagents were obtained from Gibco-BRL (Grand Island, NY, USA). Fetal bovine serum (FBS) was purchased from HyClone (Logan, UT, USA). Unless otherwise specified, small-molecule reagents and assay kits were purchased from TargetMol (Wellesley Hills, MA, USA), including curcumin (Cat. No. T1516), adenosine 5′-triphosphate (ATP; Cat. No. T20089), lipopolysaccharide (LPS; *Escherichia coli* O55:B5; Cat. No. T11855), Cell Counting Kit-8 (CCK-8; Cat. No. C0005), and NAC (Cat. No. T0875). Primary antibodies against NLRP3 and β-actin were obtained from Proteintech (Rosemont, IL, USA), whereas primary antibodies against cleaved caspase-1, cleaved IL-1β, vimentin, and E-cadherin were obtained from ABclonal (Woburn, MA, USA). For Transwell invasion assays, 24-well Transwell inserts with 8.0-μm pore polycarbonate membranes were obtained from Corning Costar (Corning, NY, USA; Cat. No. 3422), and growth factor-reduced Matrigel matrix was obtained from Corning (Corning, NY, USA; Cat. No. 354230). Other chemicals not otherwise specified were purchased from Sigma-Aldrich (St. Louis, MO, USA). Cell culture media were obtained from Thermo Fisher Scientific (Waltham, MA, USA).

### 2.2. Cell Culture

Human prostate cancer cell lines PC-3 and DU145 were obtained from the American Type Culture Collection (ATCC) (Manassas, VA, USA). PC-3 cells were maintained in F-12K Medium (Kaighn’s Modification) supplemented with 10% FBS and 1% penicillin/streptomycin (P/S). DU145 cells were cultured in RPMI-1640 supplemented with 10% FBS and 1% P/S. Cells were incubated at 37 °C in a humidified atmosphere containing 5% CO_2_, and routinely subcultured using standard trypsinization procedures. Cells were used at limited passage levels and were routinely monitored for contamination.

### 2.3. Cell Treatment and Stimulation

For inflammasome activation studies, cells were initially pretreated with either curcumin (10 or 25 μM) or NAC (2 mM) for 24 h. Following pretreatment, the culture medium was replaced with fresh medium containing 0.5 μg/mL LPS to prime the cells for another 24 h. To trigger inflammasome activation, the cells were subsequently stimulated with 5 mM ATP for 1 h.

For experiments involving cell migration and ROS production, a similar protocol was employed: cells were pre-incubated with the indicated concentrations of curcumin or NAC for 24 h prior to LPS stimulation at 0.5 μg/mL, as detailed in the respective figure legends. For RT–qPCR analysis of EMT-associated genes, cells were treated for 24 h with vehicle control, LPS (0.5 μg/mL) alone, or LPS (0.5 μg/mL) in combination with curcumin (10 or 25 μM) or NAC (2 mM), as indicated. In all experimental groups, vehicle-only treatment served as the control to account for any solvent effects. Curcumin was dissolved in DMSO as a 100 mM stock solution, and the final concentration of DMSO in the culture medium was maintained at ≤0.1% in all experiments. Vehicle-treated control groups received the corresponding concentration of DMSO alone.

### 2.4. Cell Viability Assay (CCK-8)

To evaluate the cytotoxic effects of individual treatments, PC-3 and DU145 cells were seeded at 5 × 10^3^ cells/well in 96-well plates. After overnight attachment, cells were subjected to the following treatment conditions: LPS (0.5 μg/mL) combined with ATP (5 mM), or curcumin (10, 25, 50, or 100 μM) as a single agent. These treatments were applied independently to assess their respective effects on cell viability over a 0–96 h time course. At each indicated time point, cell viability was quantified using the CCK-8 assay according to the manufacturer’s instructions. Absorbance was measured at 450 nm, and values were normalized to the untreated control group.

For viability profiling only, LPS and ATP were co-administered throughout the indicated time course to evaluate potential cytotoxicity under combined inflammatory stimulation. This approach was used to facilitate continuous time-course assessment and does not reflect the sequential priming/trigger protocol employed in inflammasome activation experiments.

### 2.5. Wound-Healing Migration Assay

**Figure 1:**
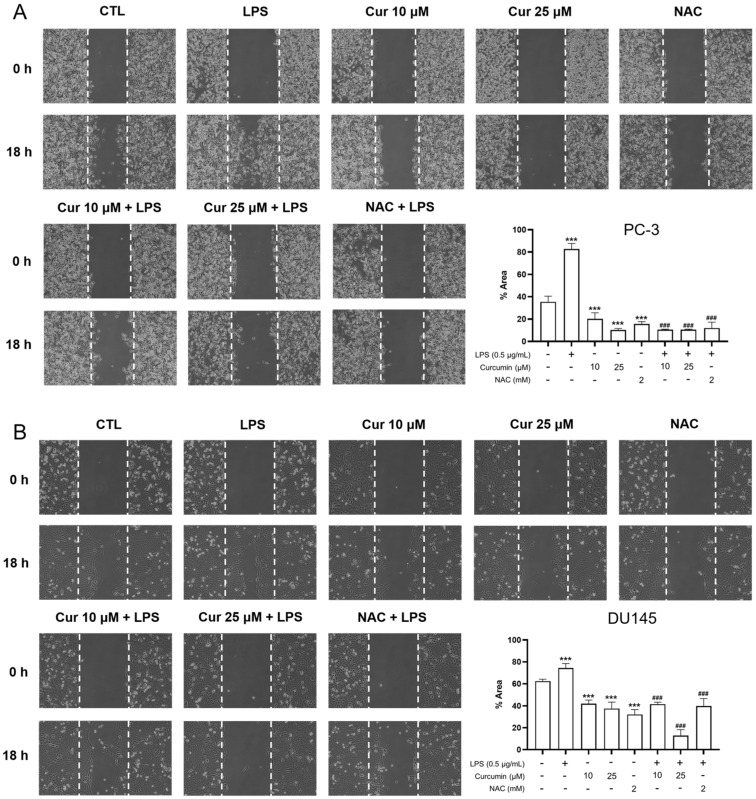
Curcumin and NAC suppress LPS-induced migration in prostate cancer cells. PC-3 (**A**) and DU145 (**B**) cells were pretreated with curcumin (Cur; 10 or 25 μM) or NAC (2 mM) for 24 h, followed by stimulation with LPS (0.5 μg/mL). Uniform cell-free gaps were generated using silicone culture inserts. The migration starting point (0 h) was defined as 24 h after insert removal to allow for cell stabilization. Subsequent images were captured 18 h after the defined 0 h time point using a Nikon Eclipse 800 microscope (100×). Dashed lines delineate the wound margins. Wound closure was quantified by measuring the remaining wound area and expressed as a percentage of the initial wound area at 0 h. Data are presented as mean ± SD from *n* = 3 independent experiments. Statistical significance was assessed using one-way ANOVA followed by Tukey’s multiple-comparisons test. *** *p* < 0.001 vs. CTL; ### *p* < 0.001 vs. LPS alone.

For the migration assay, cells were first pretreated with curcumin (10 or 25 μM) or NAC (2 mM) for 24 h, followed by stimulation with LPS (0.5 μg/mL). Following pretreatment, uniform cell-free gaps were created using silicone culture inserts. After insert removal, the monolayers were washed with PBS to remove debris and then maintained in treatment-specific fresh medium. To allow for initial cell stabilization, the starting point for migration tracking was defined as 24 h after insert removal (referred to as 0 h in Figure 1). Subsequent images were acquired 18 h later using a Nikon Eclipse 800 microscope (Nikon, Tokyo, Japan) at 100× magnification. The extent of wound closure was calculated by comparing the remaining cell-free area to the baseline area at 0 h.

### 2.6. Transwell Matrigel Invasion Assay

Cell invasion was assessed using 24-well Transwell inserts with 8.0-μm pore membranes. Growth factor-reduced Matrigel was thawed overnight at 4 °C, kept on ice, diluted to 1 mg/mL in pre-chilled serum-free medium, and 100 μL was added to each upper insert. The Matrigel-coated inserts were incubated at 37 °C for 1–2 h to allow polymerization. For drug pretreatment, PC-3 and DU145 cells were seeded in 6-well plates and incubated overnight for attachment. Cells were then treated with vehicle control, curcumin (10 or 25 μM), or NAC (2 mM) for 24 h. After pretreatment, the cells were harvested by trypsinization, washed once with PBS, and resuspended in serum-free medium. A total of 5 × 10^4^ cells in 100 μL serum-free medium were seeded into the upper chamber of each Matrigel-coated Transwell insert, and the lower chamber was filled with 600 μL complete medium containing 10% FBS as a chemoattractant. LPS (0.5 μg/mL) was then added to the designated groups, and the cells were incubated for an additional 24 h at 37 °C in a humidified incubator with 5% CO_2_. After incubation, non-invading cells and residual Matrigel on the upper surface of the membrane were gently removed with a cotton swab. Cells that had invaded the lower surface were fixed with 100% methanol for 15 min, stained with 0.1% crystal violet for 30 min, rinsed thoroughly with water, and air-dried. Representative images were captured under a light microscope at 100× magnification. Invaded cells were quantified from three randomly selected non-overlapping fields using ImageJ (version 1.53; National Institutes of Health, Bethesda, MD, USA) and expressed relative to the control group. Data from three independent experiments were used for statistical analysis.

### 2.7. Intracellular ROS Measurement by Flow Cytometry (CM-H_2_DCFDA)

Intracellular ROS production was quantified using the cell-permeable fluorescent probe CM-H_2_DCFDA (Cat. No. C6827; Thermo Fisher Scientific/Invitrogen, Waltham, MA, USA). Following the indicated 24 h treatments, cells were gently rinsed with PBS and incubated with 5 μM CM-H_2_DCFDA (diluted in PBS) for 30 min at 37 °C in the dark. After dye loading, cells were washed to remove excess probe, harvested using a cell scraper, and resuspended in PBS for immediate analysis.

**Figure 2:**
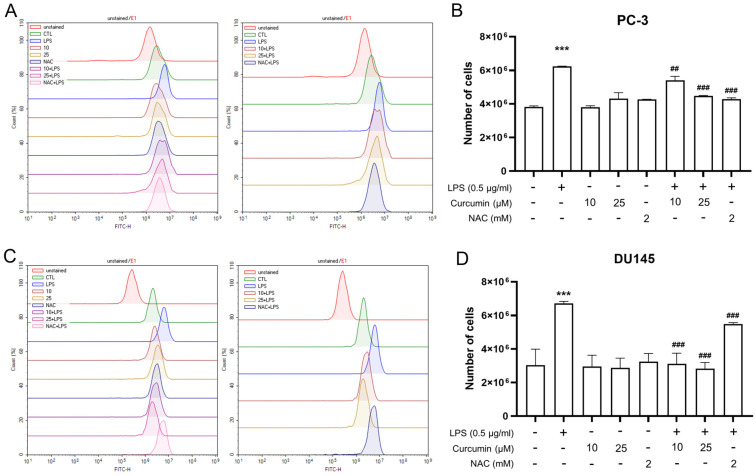
Flow-cytometric quantification of intracellular ROS using CM-H_2_DCFDA in PC-3 and DU145 cells. PC-3 (**A**,**B**) and DU145 (**C**,**D**) cells were treated with vehicle control (CTL), LPS (0.5 μg/mL) alone for 24 h, curcumin (10 or 25 μM) alone for 24 h, NAC (2 mM) alone for 24 h, or pretreated with curcumin (10 or 25 μM) or NAC (2 mM) for 24 h followed by stimulation with LPS (0.5 μg/mL) for an additional 24 h, as indicated. After treatment, cells were loaded with CM-H_2_DCFDA and analyzed by flow cytometry; DCF fluorescence was collected in the FITC-H channel. Unstained samples were used to define background fluorescence and to set the E1 gate. (**A**,**C**) Representative histograms (*y*-axis: Count (%)) of DCF fluorescence distribution. (**B**,**D**) Quantification of ROS-positive cells is presented as the number of cells within the E1 gate (*y*-axis: Number of cells). Data are shown as mean ± SD from *n* = 3 independent experiments. Statistical significance was determined by one-way ANOVA followed by Tukey’s multiple-comparisons test. *** *p* < 0.001 vs. CTL; ## *p* < 0.01 and ### *p* < 0.001 vs. LPS alone.

Flow cytometric analysis was conducted using an Agilent NovoCyte flow cytometer (Agilent Technologies, Inc., Santa Clara, CA, USA). Fluorescence was excited at 488 nm, and CM-H_2_DCFDA-derived DCF fluorescence was collected in the FITC channel (FITC-H). Data acquisition and analysis were performed using NovoExpress software (version 1.5.0, Agilent Technologies, Inc.). For each sample, at least 10,000 events were collected within the primary cell population gate defined by forward- and side-scatter (FSC/SSC). Unstained controls were used to define background fluorescence and establish the ROS-positive gate (E1). ROS output was quantified as the number of events within the E1 gate, consistent with the data presentation in Figure 2.

### 2.8. IL-1β ELISA

Secreted human IL-1β in cell culture supernatants was quantified using a Human IL-1 beta ELISA Kit (Catalog No. RK00001; ABclonal, Woburn, MA, USA). The assay uses a standard curve spanning 6.25–400 pg/mL (manufacturer’s specifications). After the indicated sequential treatments, conditioned media were collected and clarified by centrifugation at 1000× *g* for 10 min at 4 °C. Supernatants were aliquoted and stored at −80 °C until analysis, avoiding repeated freeze–thaw cycles. All reagents were equilibrated to room temperature prior to use. Standards and samples were loaded at 100 μL/well and measured in duplicate according to the manufacturer’s protocol. Absorbance was read at 450 nm using a microplate reader (Thermo Fisher Scientific, Waltham, MA, USA). IL-1β concentrations were calculated from the standard curve and corrected for dilution when applicable, and secretion levels were further normalized to cell number and expressed as pg/mL per 1 × 10^6^ cells. Data are presented as mean ± SD from *n* = 3 independent experiments.

### 2.9. RT–qPCR Analysis

Total RNA was extracted from cultured cells using TRIzol reagent according to the manufacturer’s protocol. RNA was reverse-transcribed into cDNA using the MultiScribe™ Reverse Transcriptase Kit, and quantitative PCR was performed on a Bio-Rad real-time PCR detection system (Bio-Rad, Hercules, CA, USA) using SYBR Green Master Mix; both reagents were obtained from Applied Biosystems (Foster City, CA, USA). β-Actin (ACTB) was used as the internal reference gene. Relative mRNA expression levels of SNAI1, CDH1, and VIM were calculated using the 2^−ΔΔCt^ method and are presented as fold changes relative to the control group.

Primer sequences (5′→3′) were as follows: SNAI1 (forward: TCTGAGGCCAAGGATCTCCAGGC; reverse: CAGGTTGGAGCGGTCAGCGAA), CDH1 (forward: GCCTGGGACTCCACCTACA; reverse: TCTGAGGCCAGGAGAGGAG), VIM (forward: CAGGTGGGACCAGCTAACCAA; reverse: TGCCAGACGCATTGTCA), and ACTB (forward: CACACTGTGCCCATCTACGA; reverse: CTCCTTAATGTCACGCACGA). Primer sequences were adopted from a published study [29].

### 2.10. Western Blot Analysis

Cells were harvested and lysed in RIPA buffer supplemented with a protease inhibitor cocktail on ice. Lysates were clarified by centrifugation, and total protein concentrations were determined using the Bradford assay (Bio-Rad, Hercules, CA, USA). Equal amounts of protein were mixed with loading buffer, denatured, and separated by 10% SDS–PAGE, then transferred to PVDF membranes. Membranes were blocked in PBS containing 0.1% Tween-20 (PBST) and 5% (*w*/*v*) skim milk for 1 h at room temperature, then incubated overnight at 4 °C with the designated primary antibodies. Specifically, membranes were probed with primary antibodies against NLRP3, cleaved caspase-1, cleaved IL-1β, vimentin, E-cadherin, and β-actin (loading control), followed by incubation with HRP-conjugated secondary antibodies for 1 h at room temperature after PBST washes. Immunoreactive bands were visualized using an enhanced chemiluminescence (ECL) detection system (PerkinElmer, Shelton, CT, USA). Densitometric analysis was performed using ImageJ (version 1.53). Briefly, band intensities were measured after background subtraction, and the intensity of each target protein was normalized to the corresponding β-actin band from the same lane. The normalized values were then expressed relative to the untreated control group for quantitative comparison. Representative immunoblots are shown, along with densitometric data from independent experiments.

### 2.11. Statistical Analysis

Data are presented as mean ± SD from independent experiments. For comparisons among multiple groups, one-way ANOVA followed by Tukey’s multiple-comparisons test was used. For time-course viability experiments involving two factors (treatment and time), a two-way ANOVA followed by Dunnett’s multiple-comparisons test was used to compare each treatment group with the untreated control (CTL) at the corresponding time point. A *p*-value < 0.05 was considered statistically significant.

## 3. Results

### 3.1. Selection of Sub-Cytotoxic Curcumin Concentrations for Functional Assays

**Figure 3:**
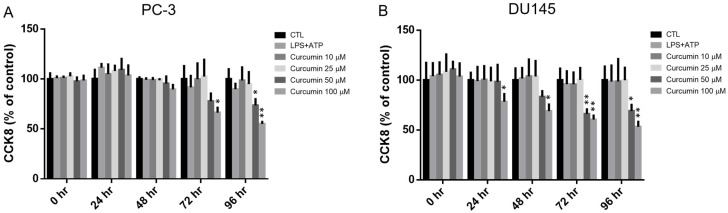
Independent effects of LPS/ATP co-treatment and curcumin on the viability of PC-3 and DU145 cells. (**A**) PC-3 and (**B**) DU145 cells were treated with either LPS (0.5 μg/mL) plus ATP (5 mM) or curcumin (Cur; 10, 25, 50, or 100 μM) for the indicated time points (0–96 h). The LPS/ATP and curcumin treatment conditions were evaluated independently. Cell viability was determined using the CCK-8 assay and expressed as a percentage of the untreated control (CTL, set to 100%). Data represent the mean ± SD from *n* = 3 independent experiments. Statistical differences were analyzed using two-way ANOVA followed by Dunnett’s multiple-comparisons test, comparing each treatment group with CTL at the corresponding time point. * *p* < 0.05, ** *p* < 0.01 vs. CTL.

To establish appropriate dosing for subsequent functional and mechanistic studies, we first evaluated the effects of LPS/ATP co-treatment and curcumin on the viability of PC-3 and DU145 cells. Using the CCK-8 assay, we monitored cell survival over a 0–96 h time course (Figure 3). LPS (0.5 μg/mL) in combination with ATP (5 mM) did not significantly compromise cell viability in either cell line at any time point. In contrast, curcumin showed a dose- and time-dependent impact on viability. In PC-3 cells, 10 and 25 μM curcumin were well-tolerated, whereas 50 and 100 μM reduced viability at later time points, particularly at 72 and 96 h. DU145 cells exhibited greater sensitivity, with 100 μM curcumin decreasing viability as early as 24 h. Based on these viability profiles, 10 and 25 μM curcumin were selected for subsequent assays to minimize confounding cytotoxicity, and NAC (2 mM) was included as an antioxidant comparator.

### 3.2. Curcumin Attenuates LPS-Induced Migratory Potential and EMT-Associated Transcriptional Reprogramming

To investigate the impact of curcumin on the metastatic potential of prostate cancer cells under inflammatory stress, we performed a standardized wound-healing assay. In both PC-3 and DU145 cell lines, stimulation with 0.5 μg/mL LPS significantly accelerated wound closure compared to vehicle-treated controls (*p* < 0.001), indicating a marked enhancement in migratory capacity (Figure 1). Notably, pretreatment with curcumin (10 or 25 μM) dose-dependently abrogated this LPS-driven motility. This inhibitory effect was reciprocated by the antioxidant NAC (2 mM), suggesting that the anti-migratory action of curcumin may be linked to the modulation of oxidative stress pathways (*p* < 0.001 vs. LPS) (Figure 1).

**Figure 4:**
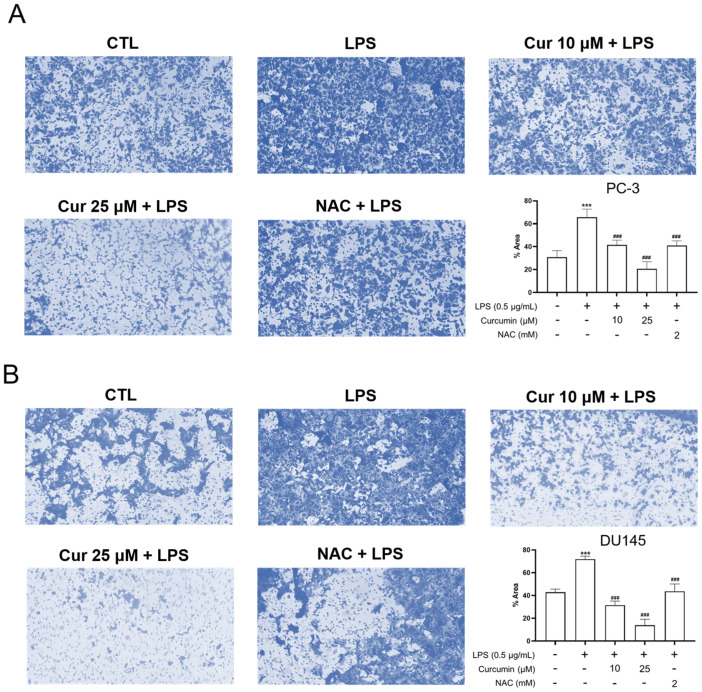
Curcumin and NAC pretreatment suppress LPS-induced invasive activity in androgen-independent prostate cancer cells. PC-3 (**A**) and DU145 (**B**) cells were pretreated in 6-well plates with curcumin (Cur; 10 or 25 μM) or NAC (2 mM) for 24 h. After pretreatment, cells were harvested and seeded into Matrigel-coated 24-well Transwell inserts (8.0-μm pore size) in serum-free medium, and then stimulated with LPS (0.5 μg/mL) for an additional 24 h. The lower chambers contained complete medium supplemented with 10% FBS as a chemoattractant. After incubation, non-invading cells on the upper surface of the membrane were removed, and invaded cells on the underside were fixed with methanol, stained with crystal violet, and photographed under a light microscope at 100× magnification. Representative images are shown. Quantification was performed from three randomly selected non-overlapping fields and expressed relative to the control group. Data are presented as mean ± SD from three independent experiments. Statistical significance was determined by one-way ANOVA followed by Tukey’s multiple-comparisons test. *** *p* < 0.001 vs. CTL; ### *p* < 0.001 vs. LPS.

To further validate curcumin’s effect on LPS-induced aggressive behavior beyond the scratch assay, we performed a Matrigel-coated Transwell invasion assay. As shown in Figure 4, LPS stimulation significantly increased invasive activity in both PC-3 and DU145 cells compared with the control group (*p* < 0.001). Pretreatment with curcumin (10 or 25 μM) markedly suppressed LPS-induced invasion, and NAC produced a comparable inhibitory effect (*p* < 0.001 vs. LPS) (Figure 4). These findings suggest that curcumin not only reduces wound closure but also suppresses LPS-induced invasive behavior in androgen-independent prostate cancer cells.

**Figure 5:**
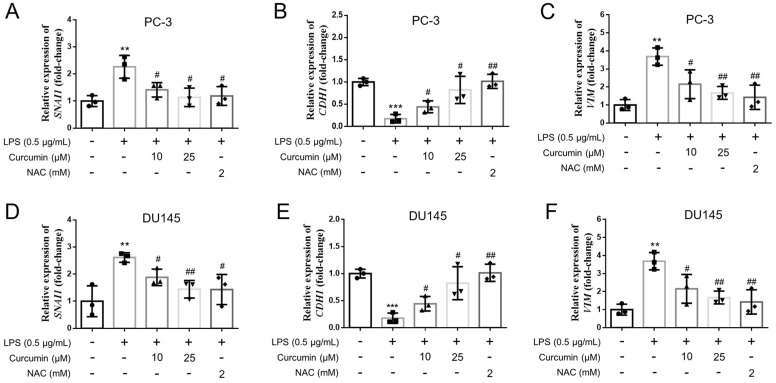
LPS alters EMT-associated gene expression and is modulated by curcumin or NAC in PC-3 and DU145 cells. PC-3 (**A**–**C**) and DU145 (**D**–**F**) cells were treated for 24 h with vehicle control (CTL), LPS (0.5 μg/mL), or LPS in combination with curcumin (10 or 25 μM) or NAC (2 mM), as indicated. Total RNA was isolated and reverse-transcribed, and the mRNA levels of *SNAI1* (Snail; (**A**,**D**)), *CDH1* (E-cadherin; (**B**,**E**)), and *VIM* (vimentin; (**C**,**F**)) were quantified by RT–qPCR. Gene expression was normalized to *ACTB* (β-actin) and calculated using the 2^−ΔΔCt^ method, with results expressed as fold-change relative to CTL. Data are presented as mean ± SD from *n* = 3 independent experiments. Statistical significance was determined by one-way ANOVA followed by Tukey’s multiple-comparisons test. ** *p* < 0.01 and *** *p* < 0.001 vs. CTL; # *p* < 0.05 and ## *p* < 0.01 vs. LPS.

Since migratory and invasive phenotypes are often associated with epithelial–mesenchymal transition (EMT)-related remodeling, we next examined the expression of key EMT-associated markers at the mRNA level. In both PC-3 and DU145 cells, LPS treatment induced EMT-associated transcriptional changes, characterized by increased expression of the mesenchymal markers SNAI1 and VIM together with decreased expression of the epithelial marker CDH1 (Figure 5). Curcumin treatment effectively reversed these changes, leading to reduced SNAI1 and VIM expression and partial restoration of CDH1 expression. NAC produced similar effects, further supporting the involvement of oxidative stress-related signaling in LPS-induced EMT-associated gene expression changes. Collectively, these findings suggest that curcumin suppresses LPS-induced motility and invasive activity, accompanied by attenuation of EMT-associated transcriptional reprogramming in androgen-independent prostate cancer cells (Figure 1, Figure 4 and Figure 5).

### 3.3. Curcumin Suppresses LPS-Induced Intracellular ROS Accumulation

The observation that the redox-modulating comparator NAC effectively mimicked the inhibitory effects of curcumin on LPS-driven migration, invasion, and EMT-related gene expression (Figure 1, Figure 4 and Figure 5) strongly suggested the involvement of oxidative stress. To test this hypothesis, we monitored intracellular ROS production using CM-H_2_DCFDA staining and flow cytometry. ROS-positive populations were precisely quantified using the E1 gate, established with unstained controls (Figure 2).

As shown in Figure 2, exposure to LPS (0.5 μg/mL) for 24 h resulted in a robust increase in the number of ROS-positive cells in both PC-3 and DU145 lines compared with vehicle-treated controls (*p* < 0.001). Pharmacological intervention with curcumin significantly blunted this oxidative surge. Specifically, in PC-3 cells, curcumin elicited a dose-dependent reduction in ROS accumulation, with 25 μM exerting a more pronounced inhibitory effect than 10 μM (*p* < 0.01 and *p* < 0.001, respectively). In DU145 cells, both concentrations of curcumin (10 and 25 μM) were equally effective in attenuating LPS-induced ROS generation (*p* < 0.001).

Consistent with its role as a redox-modulating comparator, NAC (2 mM) also significantly reduced ROS levels in response to LPS stimulation across both cell lines. Collectively, these results support an association between LPS-induced oxidative stress and the observed pro-migratory and EMT-associated responses in prostate cancer cells.

### 3.4. Curcumin Dampens LPS/ATP-Induced NLRP3-Associated Responses and IL-1β Secretion

**Figure 6:**
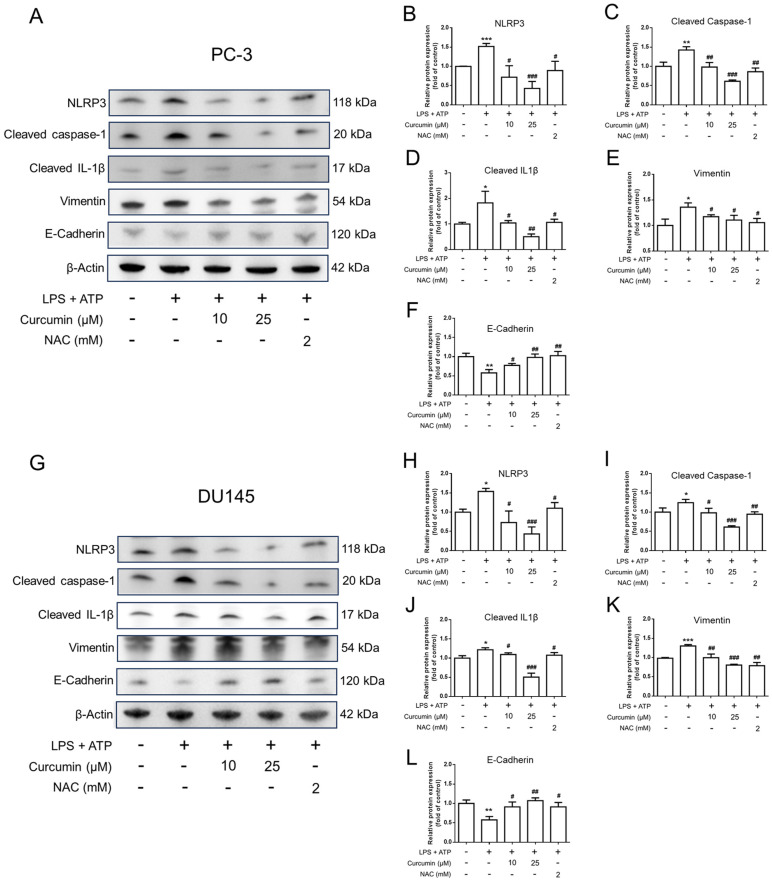
Curcumin and NAC modulate LPS/ATP-induced inflammasome-associated and EMT-related protein changes in PC-3 and DU145 cells. PC-3 cells (**A**–**F**) and DU145 cells (**G**–**L**) were pretreated with vehicle, curcumin (Cur; 10 or 25 μM), or NAC (2 mM) for 24 h, then primed with LPS (0.5 μg/mL) for an additional 24 h, followed by ATP stimulation (5 mM) for 1 h. Total cell lysates were collected and subjected to Western blot analysis using specific antibodies against NLRP3, cleaved caspase-1, cleaved IL-1β, vimentin, E-cadherin, and β-actin. Representative immunoblots are shown, and the corresponding densitometric analyses were normalized to β-actin and expressed relative to the control group. Data are presented as mean ± SD from *n* = 3 independent experiments. Statistical significance was determined by one-way ANOVA followed by Tukey’s multiple-comparisons test. * *p* < 0.05, ** *p* < 0.01, *** *p* < 0.001 vs. CTL; # *p* < 0.05, ## *p* < 0.01, ### *p* < 0.001 vs. LPS + ATP.

**Figure 7:**
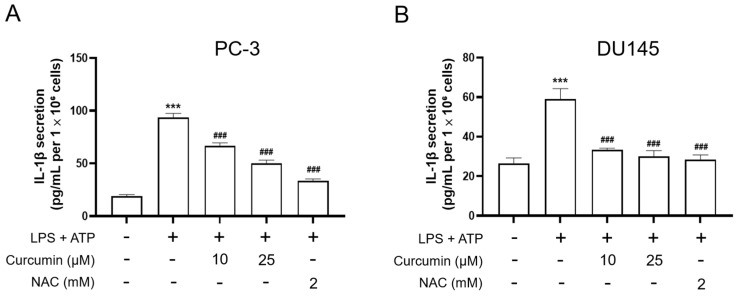
ELISA quantification of secreted IL-1β in culture supernatants from PC-3 and DU145 cells. PC-3 (**A**) and DU145 (**B**) cells were pretreated with vehicle, curcumin (10 or 25 μM), or NAC (2 mM) for 24 h, then primed with LPS (0.5 μg/mL) for an additional 24 h, followed by ATP stimulation (5 mM) for 1 h. Following ATP stimulation, cell culture supernatants were collected, and IL-1β concentrations were determined by ELISA (pg/mL). Data are presented as mean ± SD from *n* = 3 independent experiments. Statistical significance was analyzed by one-way ANOVA followed by Tukey’s multiple-comparisons test. *** *p* < 0.001 vs. CTL; ### *p* < 0.001 vs. LPS priming + ATP.

To elucidate whether the anti-motility effects of curcumin are accompanied by changes in inflammasome-associated signaling, we examined inflammasome-related and EMT-related protein expression in PC-3 and DU145 cells. Following the sequential treatment protocol—comprising a 24 h pretreatment with curcumin or NAC, a subsequent 24 h priming with LPS (0.5 μg/mL), and a final 1 h stimulation with ATP (5 mM)—total cell lysates were subjected to Western blot analysis. As shown in Figure 6, LPS/ATP stimulation increased the protein levels of NLRP3, cleaved caspase-1, cleaved IL-1β, and vimentin, while decreasing E-cadherin expression, in both PC-3 and DU145 cells. Consistent with the qPCR findings, curcumin pretreatment attenuated these changes, and NAC showed similar effects. Together, these results suggest that curcumin suppresses LPS/ATP-induced inflammasome-related responses and reverses EMT-related protein changes in androgen-independent prostate cancer cells. Mirroring the observed changes in protein expression, ELISA analysis of culture supernatants showed that the LPS-primed, ATP-triggered stimulation led to a significant surge in IL-1β secretion (*p* < 0.001 vs. CTL) (Figure 7). Notably, this inflammatory response was effectively curtailed by curcumin pretreatment, which yielded a dose-dependent reduction in IL-1β levels (*p* < 0.001 vs. LPS + ATP). Similar inhibitory trends were observed with NAC treatment, consistent with a potential contribution of redox-sensitive signaling to inflammasome-associated suppression. Collectively, these findings demonstrate that curcumin exerts a marked inhibitory effect on NLRP3-mediated inflammatory responses, effectively reducing both sensor protein expression and IL-1β secretion in prostate cancer cells.

## 4. Discussion

In this study, curcumin attenuated inflammation-driven aggressive features in androgen-independent prostate cancer cells. Taken together, the data suggest that curcumin limits both motility-associated phenotypes and inflammasome-associated inflammatory outputs under pro-inflammatory conditions. The parallel effects observed with NAC further support the idea that redox-sensitive signaling contributes to these responses. These findings identify curcumin as a potential modulator of inflammation-linked tumor-promoting processes in androgen-independent prostate cancer cells. Importantly, the contribution of the present study lies not in proposing a completely new signaling axis, but in integrating motility/invasion, EMT-associated remodeling, redox changes, and inflammasome-associated responses within a single inflammation-driven model of androgen-independent prostate cancer.

LPS stimulation promoted a clear motile phenotype in both PC-3 and DU145 cells, as shown by accelerated wound closure. This functional change was accompanied by a coordinated shift in EMT-associated transcripts, with increased SNAI1 and VIM and reduced CDH1, consistent with an EMT-like program [11,12]. These markers are widely used because they reflect a loss of epithelial adhesion and a gain of mesenchymal traits—changes that facilitate cell movement and are commonly linked to invasive and metastatic behavior [30]. Importantly, the concordance between phenotypic and molecular changes supports a biologically coherent interpretation of the response.

LPS also induced a marked increase in intracellular oxidative stress, reflected by a higher number of ROS-positive events in CM-H_2_DCFDA flow cytometry. Both curcumin and NAC significantly reduced this ROS signal, in parallel with their inhibitory effects on migration and EMT-associated gene changes. Although these data do not establish causality, they are consistent with ROS acting as a signaling node that can amplify inflammatory responses and support EMT-linked motility programs. NAC’s parallel effects support the view that redox imbalance contributes to LPS-driven plasticity.

Curcumin’s anti-migratory effect in our model fits well with what is already known about its biology. Curcumin has been widely reported to modulate NF-κB–related inflammatory signaling and cellular redox balance, and these activities have been linked to reduced motility and EMT-associated changes in prostate cancer models [31]. What our study adds is a more specific context: under an inflammation-driven stimulus (LPS), curcumin at non–growth-limiting doses (10–25 μM) was sufficient to blunt migration and normalize EMT-associated transcripts in androgen-independent PC-3 and DU145 cells. In this setting, the parallel response to NAC further supports the idea that curcumin’s impact on migration is closely tied to inflammatory redox signaling rather than non-specific cytotoxicity. Consistent with the transcript-level findings, the newly added Western blot data further supported EMT-associated remodeling, as LPS/ATP stimulation increased vimentin and reduced E-cadherin expression, whereas curcumin and NAC reversed these changes in both PC-3 and DU145 cells.

To probe inflammasome-related outputs, we used the canonical LPS priming followed by ATP triggering protocol and observed a clear induction of inflammasome-associated responses in both PC-3 and DU145 cells, including an increased NLRP3 protein signal and robust IL-1β release into the culture supernatant. Although canonical NLRP3 inflammasome activity has been most extensively characterized in immune cells, previous studies have reported that aggressive prostate cancer cell lines, including PC-3 and DU145, express inflammasome-related components and can exhibit inflammasome-associated signaling under defined experimental conditions [32,33]. Therefore, in the present study, these cells were used to examine inflammasome-associated responses in a prostate cancer context rather than to model the full canonical immune-cell inflammasome setting. Curcumin pretreatment significantly reduced IL-1β secretion, a functional downstream output of this pathway, and the representative immunoblots showed a concomitant reduction in NLRP3 signaling. Although we now include cleaved caspase-1 and cleaved IL-1β as additional inflammasome-associated protein readouts, we did not evaluate ASC oligomerization/speck formation; therefore, our conclusions are best interpreted as evidence of inflammasome-associated signaling rather than definitive proof of full canonical inflammasome assembly. Similar LPS+ATP–driven cytokine induction has also been reported in prostate cancer models, supporting the relevance of this stimulation paradigm in the prostate cancer context [34].

Overall, our data support a working model in which inflammatory stimulation promotes an ROS-dependent EMT/migration program and, under LPS priming plus ATP triggering, induces inflammasome-associated IL-1β release. Curcumin dampened both arms of this response—reducing ROS-positive events alongside migration/EMT changes, and suppressing NLRP3 signal and IL-1β secretion—consistent with its known ability to modulate redox balance and inflammatory signaling. The fact that NAC produced similar effects across these assays strengthens the link between redox imbalance and these inflammation-driven phenotypes. Mechanistically, curcumin may act at the level of priming pathways (e.g., NF-κB-dependent gene induction) and/or upstream ROS generation, thereby limiting downstream plasticity and cytokine output.

Several features support the robustness of the present findings. First, we defined sub-cytotoxic curcumin doses using a time-course viability analysis (Figure 3), reducing the likelihood that downstream effects reflect non-specific growth inhibition. Second, key findings were reproduced in two androgen-independent prostate cancer cell lines (PC-3 and DU145), improving generalizability. Finally, we combined functional readouts (migration and IL-1β secretion) with molecular measurements (EMT-related transcripts, ROS by flow cytometry, and NLRP3 by immunoblot), providing convergent evidence for curcumin’s effects across phenotype and signaling outputs.

This study also has limitations. First, although the LPS priming/ATP trigger protocol increased NLRP3 signaling and IL-1β release, we also detected cleaved caspase-1 and cleaved IL-1β as additional inflammasome-associated protein readouts; however, we did not evaluate higher-order inflammasome assembly, such as ASC speck formation or oligomerization. Therefore, our conclusions are best interpreted as evidence of inflammasome-associated signaling rather than definitive proof of full canonical inflammasome assembly. Second, wound-healing assays can be influenced by proliferation; while we minimized this concern by working at sub-cytotoxic curcumin doses, follow-up studies could include mitomycin C controls or time-lapse tracking to better separate migration from growth. Third, CM-H_2_DCFDA provides a useful readout of oxidative stress but is not specific for ROS sources; complementary assays (e.g., MitoSOX for mitochondrial ROS, or antioxidant enzyme/activity measurements) would strengthen the redox interpretation. CM-H_2_DCFDA is a useful but non-specific indicator of intracellular oxidative stress, and NAC should be interpreted as a redox-modulating comparator rather than as definitive proof of ROS causality. Therefore, the present data most appropriately support an association between oxidative stress and the observed phenotypes, rather than identifying a specific ROS species or source [35]. Finally, we did not use pathway-specific inhibitors or genetic perturbations (e.g., TLR4, NF-κB, or NOX blockade) to pinpoint the upstream nodes through which curcumin acts, which remains an important next step. An important limitation of this study is the translational relevance of the curcumin concentrations used. The tested micromolar range (10–25 μM) exceeds levels typically achievable in vivo because of curcumin’s poor absorption and rapid metabolism. Therefore, these findings should be interpreted primarily as mechanistic in vitro evidence rather than as a direct indication of clinically achievable efficacy. Future studies using more physiologically relevant exposure conditions or improved formulations are warranted [31,36]. Another important limitation is the biological relevance of the LPS stimulation model. In this study, LPS was used as a defined experimental inflammatory stimulus to probe inflammation-responsive signaling and phenotypes in prostate cancer cells. However, bacterial LPS exposure is unlikely to be the predominant physiological driver in most prostate cancer contexts, and the responses observed here should therefore be interpreted within the context of an in vitro inflammatory challenge model [7].

In conclusion, curcumin suppresses inflammation-driven aggressive phenotypes in androgen-independent prostate cancer cells, including LPS-induced migration/EMT changes and LPS/ATP-associated IL-1β release, supporting its potential as a modulator of inflammation-linked tumor progression.

## Data Availability

The original contributions presented in this study are included in the article. Further inquiries can be directed to the corresponding authors.
